# Follicular Conjunctivitis due to *Chlamydia felis*—Case Report, Review of the Literature and Improved Molecular Diagnostics

**DOI:** 10.3389/fmed.2017.00105

**Published:** 2017-07-17

**Authors:** Juliana Wons, Ralph Meiller, Antonio Bergua, Christian Bogdan, Walter Geißdörfer

**Affiliations:** ^1^Augenklinik, Universitätsklinikum Erlangen, Erlangen, Germany; ^2^Mikrobiologisches Institut, Klinische Mikrobiologie, Immunologie und Hygiene, Friedrich-Alexander-Universität (FAU) Erlangen-Nürnberg, Universitätsklinikum Erlangen, Erlangen, Germany

**Keywords:** *Chlamydia felis*, *Chlamydia trachomatis*, follicular conjunctivitis, eye infection, cat flu, PCR

## Abstract

A 29-year-old woman presented with unilateral, chronic follicular conjunctivitis since 6 weeks. While the conjunctival swab taken from the patient’s eye was negative in a *Chlamydia (C.) trachomatis*-specific PCR, *C. felis* was identified as etiological agent using a pan-*Chlamydia* TaqMan-PCR followed by sequence analysis. A pet kitten of the patient was found to be the source of infection, as its conjunctival and pharyngeal swabs were also positive for *C. felis*. The patient was successfully treated with systemic doxycycline. This report, which presents one of the few documented cases of human *C. felis* infection, illustrates that standard PCR tests are designed to detect the most frequently seen species of a bacterial genus but might fail to be reactive with less common species. We developed a modified pan-*Chlamydia*/*C. felis* duplex TaqMan-PCR assay that detects *C. felis* without the need of subsequent sequencing. The role of chlamydiae-specific serum antibody titers for the diagnosis of follicular conjunctivitis is discussed.

## Introduction

Follicular conjunctivitis is characterized by conjunctival hyperemia and lymphoid follicle formation on the conjunctiva of the eyelid, which causes irritation and a red eye, often with symptoms for several weeks. A chronic follicular inflammation of the conjunctiva can be sign of a bacterial or viral infection (1). Chlamydiae are special agents, because they are obligate intracellular bacteria and cannot be cultured on standard media. Therefore, microbiological routine diagnostics relies mainly on DNA-based PCR tests that are used for detection of these bacteria in thoroughly taken, cell-containing conjunctival swabs (2). Nearly all cases of chlamydial conjunctivitis are caused by *Chlamydia* (*C*.) *trachomatis* (serotypes D to K), generally in the context of a genital infection. *C. felis*, an agent of the feline “cat flue” syndrome, is a known, but rare agent of human follicular conjunctivitis with five documented cases (3–7). The role of *C. psittaci* and *C. pneumoniae* in follicular conjunctivitis is less established because only isolated cases have been reported (6, 8).

Here, we report a human case of follicular conjunctivitis due to *C. felis* with clear evidence for transmission from a pet cat, discuss the limitations of laboratory diagnostics, and offer a newly developed real-time PCR method for the specific detection of *C. felis*.

## Case Report

**Figure 1:**
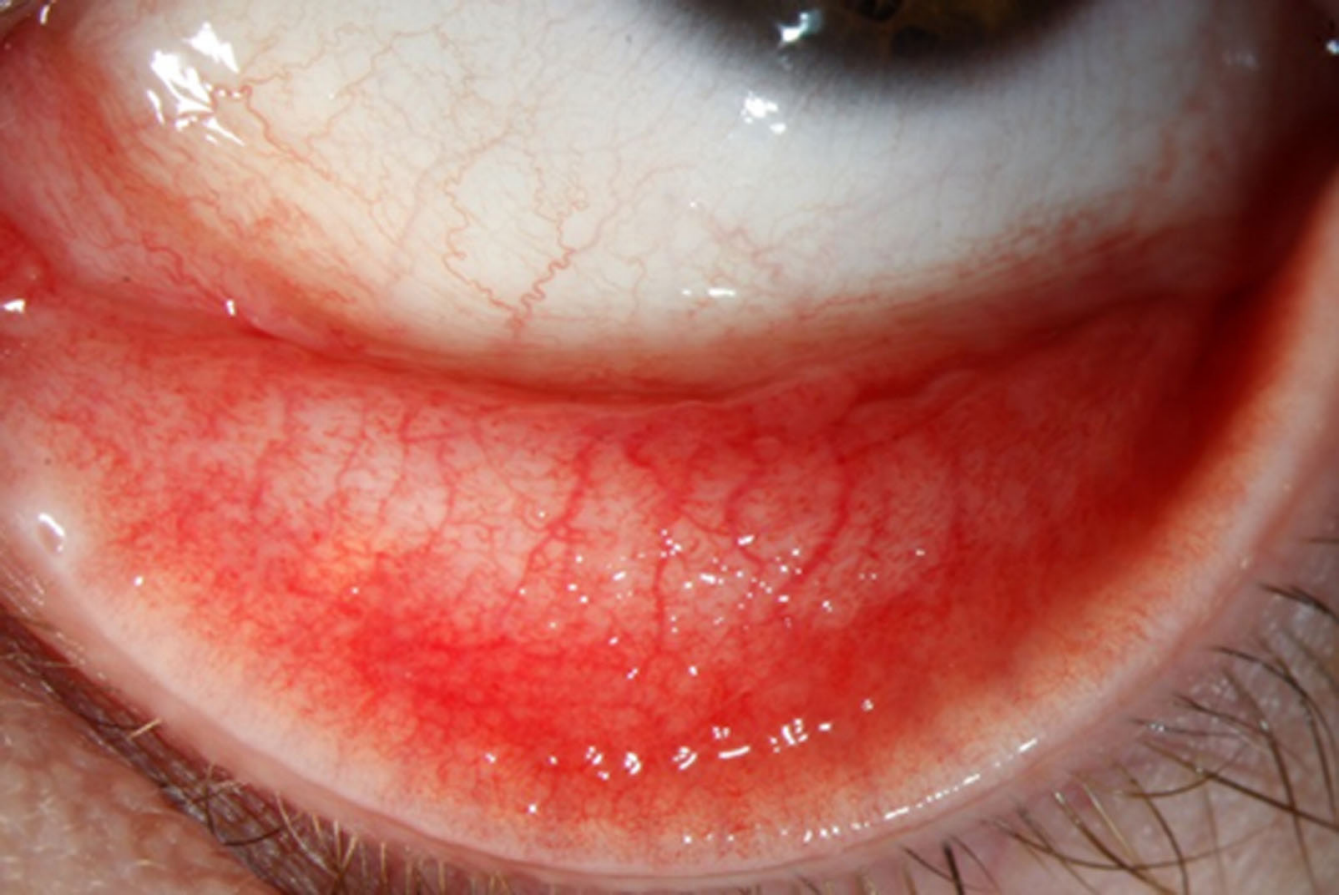
Follicular conjunctivitis in the conjunctiva tarsi of the patient’s left eye.

A 29-year-old woman presented to our outpatient clinic with a history of a reddish, swollen left eye for 6 weeks (Figure 1). She reported to suffer from severe irritation of the eye with purulent discharge in the morning. The patient was otherwise healthy and did not use any medication. She did not report any previous eye infections, systemic diseases or ophthalmologic treatments. From time to time she has used artificial tear fluids to treat a mild dry eye syndrome. The dry eye symptoms were aggravated by the daily computer work during her office job. About two months ago, she had adopted two pet kittens (cat 1 and cat 2) from an animal shelter. Whereas cat 1 soon had died from severe “cat-flu,” cat 2 was rather healthy showing only mild conjunctivitis. The patient remembered that cat 2 had sneezed into her left eye prior to the onset of her disease manifestations.

**Figure 2:**
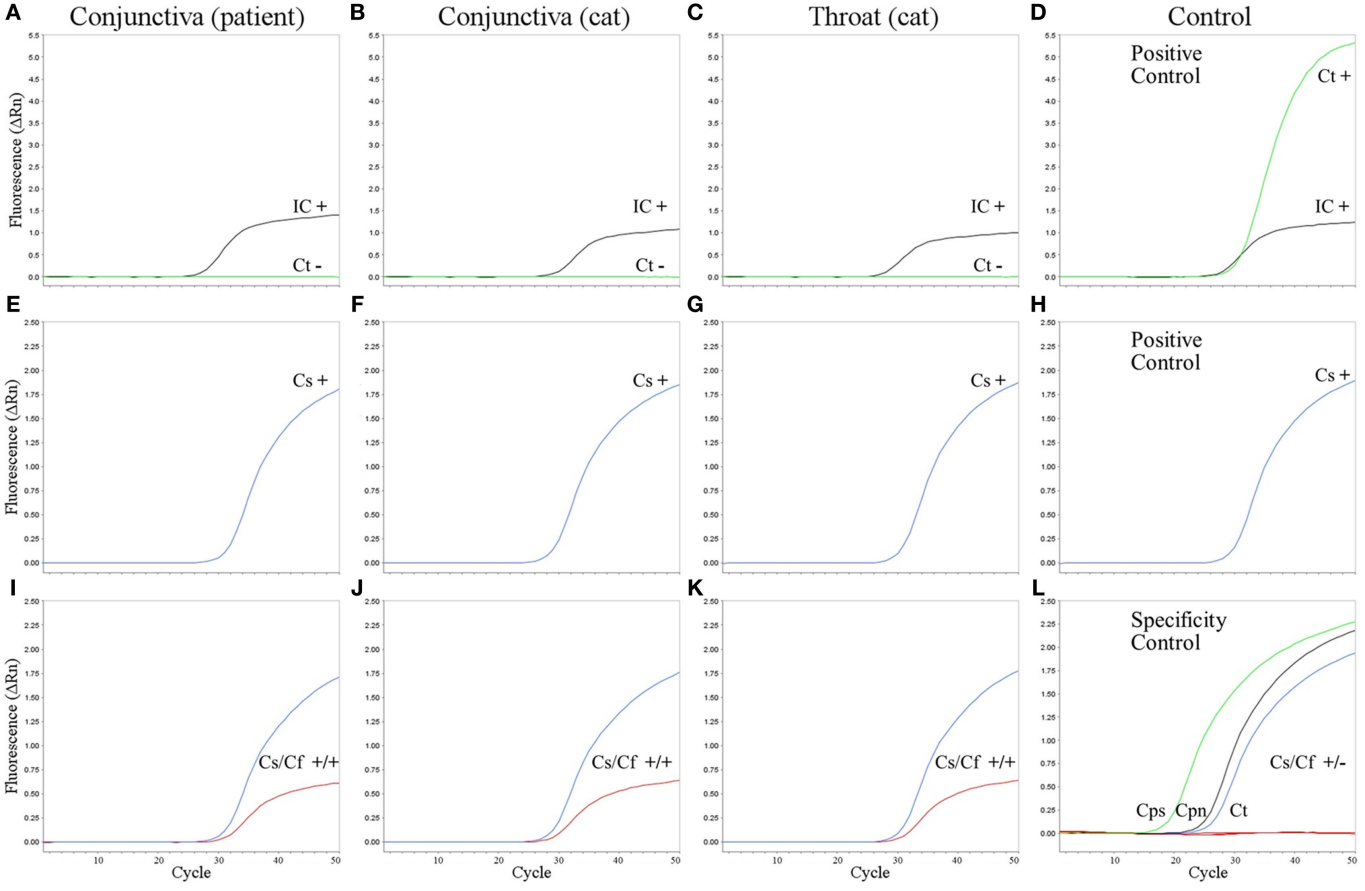
Amplification plots of real-time PCR assays. *C. trachomatis* PCR **(A–D)**, pan-*Chlamydia* PCR **(E–H)**, and new pan-*Chlamydia*/*C. felis* duplex PCR assay **(I–L)** on the conjunctival swab from the patient **(A,E,I)**, conjunctival swab from the cat **(B,F,J)**, and throat swab from the cat **(C,G,K)**. **(D,H)** Positive controls. Results (+, positive; −, negative) are given within the panels for internal control (IC, black), *C. trachomatis* (Ct, green), pan-*Chlamydia* PCR (Cs, blue), and the pan-*Chlamydia*/*C. felis* duplex PCR (Cs/Cf) containing both the genus-specific (Cs, blue) and the *C. felis*-specific probe (Cf, red). **(L)** Specificity controls of the new pan-*Chlamydia*/*C. felis* duplex PCR showing positive results with the genus-specific probe (green, black, and blue), but no cross-reactivity of the *C. felis*-specific probe (red). Three reactions are combined in one panel: DNA from *C. psittaci* (Cps, cell culture, 10^5^ infection forming units per PCR), *C. pneumoniae* (Cpn, cell culture, 2 × 10^4^ genomic copies per PCR), and *C. trachomatis* (Ct, from urethral swab specimen, 4 × 10^3^ infection forming units per PCR).

Ophthalmological examination of the patient revealed a best corrected visual acuity of 20/20 on both eyes. A follicular inflammation of the conjunctiva tarsi of the upper and lower lid of the left eye as well as a mild conjunctivitis on the right eye was found (Figure 1). The cornea of both eyes was unaffected; in particular, there were no signs of scarification or edema. There was also no evidence for an intraocular inflammation, and the retina and vitreous body were without pathological findings. Microbiological cultures of conjunctival swabs taken from both eyes yielded the growth of normal bacterial flora, but not of pathogenic bacteria or of fungi. A serum sample of the patient showed only low-level titers of antibodies against the genus *Chlamydia*, consistent with a previous chlamydial infection. Another conjunctival swab taken from the left eye was used for molecular microbiological diagnostics. The *C. trachomatis*-specific PCR was negative, but *C. felis*-DNA was detected using a pan-*Chlamydia* PCR/sequencing approach (Figure 2). The diagnosis was confirmed by PCR-based detection of a second *C. felis*-specific gene sequence (*hflX*).

To verify that the patient’s kitten (cat 2) was the source of infection, swabs from the throat and conjunctiva of the cat were tested, yielding again positive results in the pan-*Chlamydia* PCR; the subsequent sequence analysis of the PCR product led to unambiguous identification of *C. felis* (Figure 2).

After therapy with azithromycin eye drops (twice daily for 3 days) and subsequent systemic therapy with doxycycline (100 mg twice daily for 14 days), symptoms and clinical signs initially improved in our patient. However, after discontinuation of the antibiotic treatment, the conjunctival inflammation relapsed requiring a second 14 days-cycle of doxycycline treatment (as above) leading to a complete regression of the follicular conjunctivitis. Dexamethasone eye drops (five times daily for 5 weeks) were prescribed. The cat was also treated with systemic tetracycline to avoid a re-infection of the patient.

## Laboratory Diagnostic Tests and Results

### Culture

Cultures from conjunctival swabs were performed according to microbiological standard procedures on blood agar, chocolate agar and ENDO agar (37°C) as well as on Sabouraud agar (28°C) for 2 days (2). Only few colonies of coagulase-negative staphylococci were obtained.

### Indirect Immunofluorescence Assay

*Chlamydia* microimmunofluorescence test (FOCUS Diagnostics, Cypress, USA) was used for detection of combined IgG and IgM. The test contains antigens from *C. pneumoniae, C. trachomatis*, and *C. psittaci*, but does not include *C. felis*. In the patient’s serum antibodies reactive with all three *Chlamydia* species were found (titer: 1:40 each, cutoff 1:20).

### Molecular Methods

#### Isolation of DNA

The patient’s conjunctival swab used for molecular diagnostics was sent to the laboratory in 1 ml of Mastazyme^®^ *Chlamydia* transport medium (Mast Diagnostica, Reinfeld, Germany). DNA was prepared from 200 µl transport medium using the High Pure PCR Preparation Kit (Roche Diagnostics, Mannheim, Germany). Prior to DNA preparation, 150,000 copies of bacteriophage *Lambda* DNA (New England Biolabs, Frankfurt, Germany) were added to the sample for internal inhibition control. Swabs taken from the cat’s throat and conjunctiva were treated identically.

#### Real-time PCR for *C. trachomatis* and *Chlamydia* Species

Real-time PCR for specific detection of *C. trachomatis* cryptic plasmid was performed as described by Jaton et al. (9). TaqMan^®^ Gene Expression Master Mix (Life Technologies, Darmstadt, Germany) was used for PCR with 50 cycling steps (15 s 95°C and 1 min 60°C each) on a StepOne^®^ PCR System (Life Technologies, Darmstadt, Germany). Amplification of bacteriophage *Lambda* DNA indicated absence of PCR inhibitors for the three tested specimens, but no *C. trachomatis* DNA was detected (Figures 2A–C).

Pan-*Chlamydia* PCR for detection of an 23S rRNA gene fragment of *Chlamydia* spp. was performed under the same conditions as *C. trachomatis* PCR, but primers (TQF, TQR) and probe TQP were used as described (10). The conjunctival swabs from the patient and her cat as well as the throat swab from the cat tested positive for *Chlamydia* species (Figures 2E–G). Subsequent sequence analysis revealed the same sequence for all three specimens (identical to GenBank NR_076260, bp 516–646). Blast analysis identified the respective bacteria unambiguously as *C. felis*.

**Figure 3:**
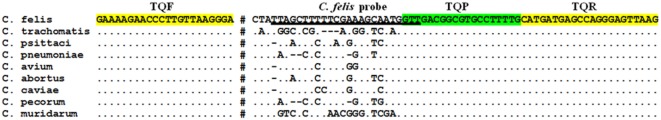
23S rRNA gene fragment as target for the pan-*Chlamydia* PCR. The primers (TQF and TQR) are highlighted in yellow, and the pan-*Chlamydia* probe (TQP) in green. The DNA sequence used as *C. felis*-specific probe (this study) is underlined. Note that despite the overlap of the two probes, there is no interference in detection because they bind to complementary DNA strands. 44 bp of conserved sequences are blanked out (marked by #). GenBank accession numbers for the aligned sequences are: *C. felis* (NR_076260, bp 516–646), *C. trachomatis* (NR_103960), *C. psittaci* (NR_102574), *C. pneumoniae* (NR_076161), *C. avium* (NR_121988), *C. abortus* (NR_077001), *C. caviae* (NR_076195), *C. pecorum* (NR_103180), *C. muridarum* (NR_076163).

A modification of the pan-*Chlamydia* PCR including *C. felis*-specific probe “CFP” (VIC-TTAGCTTTTTCGAAAGCAATGGTT-NFQ-MGB) was developed in this study (Figure 3). Using both probes (TQP and CFP) in the same PCR reaction, *C. felis* was detected in the conjunctival swab of the patient as well as in the two samples of the cat (Figures 2I–K) without any shift in sensitivity compared to PCR with TQP alone (Figures 2E–G). 12 copies of a positive control plasmid per PCR reaction reliably gave a positive result (not shown). The species-specificity of the *C. felis* probe was tested by analyzing the three pathogenic Chlamydiae most frequently found in humans: *C. trachomatis, C. psittaci*, and *C. pneumoniae* (Figure 2L). Even in the presence of high amounts of DNA of these chlamydial species, the *C. felis*-PCR did not yield a positive signal.

#### Analysis of the *C. felis hflX* Gene Sequence

To further prove the identity of *C. felis* detected in the patient and her cat, we attempted to amplify additional *C. felis* genes according to a published multilocus sequence typing approach (11). Presumably due to the low amount of chlamydial DNA and the accompanying host DNA, we only succeeded in the amplification of the *hflx* gene in the DNA prepared from the conjunctival swab of the patient (primers YPhflX5: GCTTCTARRGTACTTTTAAATG and YPhflX6: ATWTTAGAGATCTTTGCTAGYCG). The respective sequence (460 bp) was identical to the *hflX* gene from *C. felis* Fe/C-56 (GenBank AP006861, bp 883,438–883,897) and *C. felis* 11E3-3 (GenBank HE795925) and differed in 1 bp from a third reported *C. felis* sequence (11E3-2, GenBank HE795924). This result confirms the identification of *C. felis*, showing 65 bp difference to *C. caviae* (GenBank AE015925) as the fourth matching sequence.

## Discussion

### Taxonomy of *Chlamydia felis*

The pathogen known as *C. felis* today was originally termed “feline pneumonitis agent” following its first isolation from cats with upper respiratory tract disease (12). Later, the feline chlamydial isolates were grouped into the species *Chlamydia psittaci* (13). The separation of Chlamydiae into two genera (*Chlamydia* and *Chlamydophila*) on the basis of 16S rRNA sequences (14) led to the species “*Chlamydophila felis*,” which, however, was never accepted by the scientific community and caused some confusion (14, 15). Meanwhile, the proposal to reunify all Chlamydiae into one genus (*Chlamydia*) has been published (16).

### Serology in *Chlamydia* Diagnostics

In the case of chlamydial infections the diagnostic value of serology is hampered by several obstacles: (1) antibodies against different *Chlamydia* species show a high degree of cross-reactivity; (2) a high seroprevalence is found in the community as a consequence of frequent upper respiratory tract or genital infections with *C. pneumoniae* or *C. trachomatis*; and (3) the intracellular habitat of the pathogen and the mostly locally restricted epithelial infection may lead to limited antibody production (2). Therefore, in cases of uncomplicated genital infection or follicular conjunctivitis due to *C. trachomatis*, serological testing is not recommended. To assess the value of the immunofluorescence assay for cases of conjunctivitis by *C. trachomatis*, we retrospectively analyzed the serological results that were available for 24 out of the 32 cases diagnosed in our laboratory by PCR from conjunctival swabs (Table S1 in Supplementary Material). The antibody titers were highly indicative for *C. trachomatis* infection in 11 cases; consistent with *C. trachomatis* infection but showing high cross-reactive titers in 4 cases; and not indicative for a *C. trachomatis* infection in 9 cases. Thus, in uncomplicated cases of *C. trachomatis* infection such as conjunctivitis serological testing is able to confirm some cases, but is definitely not the method of choice because of its insufficient specificity and sensitivity. Negative serology does not rule out infection, and high titers still require confirmation by PCR.

To date, there is no immunofluorescence assay available for the diagnosis of human *C. felis* infections. In addition, antibody production in response to *C. felis* may be lower compared to *C. trachomatis*, because in general the infection will only affect the ocular conjunctiva, i.e., concurrent genital infection will not be present. In the case of our patient, low titers of cross-reactive, pan-*Chlamydia* antibodies, compatible with previous chlamydial infections, were detected. However, as *C. felis* antigen is not included in the microimmunofluorescence assay, definitive statements on the extent of anti-*C. felis* antibody responses cannot be made.

### Direct Detection of Chlamydiae

As Chlamydiae cannot be cultivated on standard cell-free media, they are traditionally detected by microscopical examination of stained clinical specimens or cell cultures. These procedures are laborious and need great experience. As the specificity is dependent on the staining protocol and sensitivity is lower compared to PCR (2, 17), we did not apply these techniques in our case.

### Molecular Detection of *Chlamydia* DNA

As PCR protocols show an outstanding sensitivity for detection of Chlamydiae in clinical specimens, they have almost completely replaced microscopic and cell culture procedures in routine diagnostics (2, 17). Another milestone was the development of real-time PCR protocols with the “closed-tube concept,” i.e., the tubes remain closed after PCR, thereby reducing the risk of DNA contaminations. The high sensitivity and specificity of the real-time PCR even allows screening of young women in low prevalence collectives for inapparent genital *C. trachomatis* infections (18).

The high specificity of PCR protocols always bears the risk of false negative results if mutations in the target sequence occur. To ensure the detection of putative, new plasmid variants of *C. trachomatis*, we routinely tested samples by a pan-*Chlamydia* PCR in addition to the *C. trachomatis*-specific PCR. In the case described here, the pan-*Chlamydia* PCR led to the detection of *C. felis*. As the identification of the respective species with this procedure needs laborious sequencing of the PCR product, we included a new *C. felis*-specific probe, which directly indicates the presence of *C. felis* by an additional separate amplification curve. This pan-*Chlamydia*/*C. felis* duplex PCR assay showed an unaltered performance for detection of any *Chlamydia* species (Figures 2E–K), whereas the *C. felis*-specific probe showed no cross-reactivity to other chlaymidae (Figure 2L) and succeeded in detection of *C. felis* in the patient’s and the cat’s specimens without any problem. The amplified region of the 23S rRNA gene seems to be invariable within *C. felis*, as all five sequences present in GenBank show the same sequence as also found in our patient and her cat. In addition, even if such a hypothetical variant existed, it would still be detected by the pan-*Chlamydia* probe. Species identification would then have to be done by sequence analysis of the PCR product, but with the pan-*Chlamydia*/*C. felis* duplex PCR, no chlamydial species can be missed. Thus, we believe that this method is a valuable and reliable tool for modern diagnostic laboratories.

### *C. felis* Infections in Cats and Humans

*C. felis* causes conjunctivitis and upper respiratory tract infections in cats, particularly kittens, and can be isolated in up to 30% of affected cats. Coinfections with other agents like feline herpesvirus 1, feline calicivirus, *Bordetella bronchiseptica*, and *Mycoplasma felis* lead to the “cat flu” syndrome (19, 20). In healthy cats, prevalence of *C. felis* is lower than 3% (21). Transmission within the cat population occurs through close contact, and ocular fluids are the most important source of infection. *C. felis* may also be spread by sneezing, which apparently was the route of transmission to our patient. *C. felis* is probably adapted to cats as its natural host, which offers an explanation that humans are not readily susceptible to infection unless they are exposed to high pathogen loads as it might occur by direct sneezing into a patient’s eye.

Case reports on human infections by *C. felis* do not indicate that immunosuppression is a prerequisite for infection (Table 1). Our patient also did not suffer from any underlying disease. In general, acute conjunctivitis is a frequent human condition, with an estimated six million cases per year in the USA and Chlamydiae being the cause in 1.8–5.6% of all cases (1). However, only few human cases of *C. felis* conjunctivitis have been reported (Table 1), despite its high incidence in cats and the large number of pet cats in our society. In our hospital setting, 697 conjunctival swab samples were tested for *C. trachomatis* conjunctivitis within a 7 year period (unpublished results); 32 cases of *C. trachomatis* were identified. Out of the 697 specimens, 554 were in parallel tested by a pan-*Chlamydia*-PCR, but only 1 case of *C. felis* was found. These findings strongly suggest that the incidence of *C. felis* as cause of human conjunctivitis (one of 554 patients, i.e., 0.2%) is much lower than that of *C. trachomatis* (32 of 697 cases, i.e., 4.7%), but also underline the need for a reliable and simple method for the detection of *C. felis* as described above (see Molecular Detection of *Chlamydia* DNA).

**Table 1:** Published reports on human infections by *Chlamydia felis*.

Clinical manifestation of the infection	Diagnostic evidence for the chlamydial infection	Immunosuppression	Cat contact	Reference
Conjunctivitis	Culture from conjunctiva	No	Yes	(3, 4)
Conjunctivitis, keratouveitis	Culture from conjunctiva	No	Yes	(5)
Malaise, cough	Serology only	Corticosteroids (kidney transplantation)	Yes	(22)
Endocarditis, glomerulonephritis	Serology only	No	Yes	(23)
Atypical pneumonia	Serology only	No	Yes	(24)
Conjunctivitis (2 cases)	*ompA* gene PCR and sequence analysis from conjunctiva	unknown	Yes	(6)
Conjunctivitis	Culture from conjunctiva, *omp2* gene PCR and sequence analysis from isolate	HIV	Yes	(7)
Unknown (3 cases)	16S rRNA gene PCR and sequence analysis from respiratory sample	unknown	unknown	(25)
Community-acquired pneumonia (2 cases)	Serology only	No	unknown	(26)
Conjunctivitis	23S rRNA gene PCR and sequence analysis from conjunctiva	No	Yes	This case

In accordance with previous case reports (see Table 1), our patient was successfully treated with doxycycline. Tetracyclines are also the drugs of choice in *Chlamydia*-infected cats, in which 4 weeks of therapy are recommended to achieve pathogen elimination (20).

## Conclusion

*C. felis* is a rare agent of chronic follicular conjunctivitis in humans and is acquired by close contact to infected cats via ocular fluid or sneezing. Patients with follicular conjunctivitis should be questioned about contact to cats, and if the patient’s conjunctival swab tested negative for *C. trachomatis* by PCR, the diagnostic laboratory should be asked to perform additional tests for Chlamydiae other than *C. trachomatis* or to transfer the specimen to a specialized laboratory. *Chlamydia*-infected pet animals are a potential source of infection for humans and therefore should receive curative antibiotic treatment.

## Ethics Statement

The written informed consent was obtained from the patient.

## Author Contributions

JW, RM, and AB obtained the patient’s clinical history and performed the medical examination and the treatment. CB substantially contributed to the acquisition of specimens from the cat and to analysis and interpretation of results. WG conducted the entire laboratory diagnostics and developed the respective assays. WG, JW, and CB wrote the manuscript. AB and RM critically revised the paper. All the authors have approved the final version of the manuscript for publication and agreed to be responsible for their part of the work.

## Conflict of Interest Statement

The authors declare that the research was conducted in the absence of any commercial or financial relationships that could be construed as a potential conflict of interest.
