# Supplementary material for: Follicular Conjunctivitis due to Chlamydia felis—Case Report, Review of the Literature and Improved Molecular Diagnostics
Source: Front Med (Lausanne). 2017 Jul 17;4:105. doi: 10.3389/fmed.2017.00105 (PMC5512277; doi:10.3389/fmed.2017.00105)
Supplement: Supplementary file 1 [file Table_1.DOCX]

Supplementary Material

Follicular conjunctivitis due to *Chlamydia felis* - case report, review of the literature and improved molecular diagnostics

Juliana Wons, Ralph Meiller, Antonio Bergua, Christian Bogdan, Walter Geißdörfer^*^

*** Correspondence:** Corresponding Author: walter.geissdoerfer@uk-erlangen.de

Supplementary Table 1. Serum antibody titers as determined by microimmunofluorescence assays in patients with PCR detection of *C. trachomatis* DNA in conjunctival swabs.

| **Serum sample no. / year** | ***C. pneumoniae**** | ***C. trachomatis**** | ***C. psittaci**** |
| --- | --- | --- | --- |
| indicative for *C. trachomatis* infection | |  |  |
| SE6455/2011 | < 1:10 | 1:640 | < 1:10 |
| SE7813/2012 | 1:80 | 1:640 | 1:10 |
| SE4995/2013 | < 1:10 | 1:160 | < 1:10 |
| SE5220/2013 | 1:20 | 1:320 | 1:20 |
| SE2987/2014 | 1:320 | 1:5120 | 1:80 |
| SE1921/2015 | 1:80 | 1:1280 | 1:80 |
| SE3272/2015 | < 1:10 | 1:2560 | < 1:10 |
| SE4982/2015 | 1:160 | 1:2560 | < 1:10 |
| SE6823/2015 | 1:40 | 1:640 | 1:10 |
| SE1128/2016 | < 1:10 | 1:640 | < 1:10 |
| SE5113/2016 | < 1:10 | 1:160 | < 1:10 |
|  |  |  |  |
| consistent with *C. trachomatis* infection | |  |  |
| SE2687/2010 | 1:640 | 1:640 | < 1:10 |
| SE4748/2013 | 1:160 | 1:320 | < 1:10 |
| SE1991/2014 | 1:320 | 1:320 | < 1:10 |
| SE4720/2016 | 1:160 | 1:160 | < 1:10 |
|  | |  |  |
| not indicative for *C. trachomatis* infection | |  |  |
| SE4062/2011 | 1:160 | 1:40 | 1:10 |
| SE4551/2011 | 1:1280 | 1:160 | 1:160 |
| SE5419/2013 | 1:640 | 1:40 | < 1:10 |
| SE5931/2013 | 1:20 | 1:20 | 1:10 |
| SE6074/2013 | 1:20 | 1:40 | 1:10 |
| SE4404/2014 | 1:160 | 1:80 | 1:20 |
| SE4047/2016 | 1:20 | < 1:10 | < 1:10 |
| SE6930/2016 | 1:40 | 1:10 | < 1:10 |
| SE1172/2017 | < 1:10 | < 1:10 | < 1:10 |
|  |  |  |  |
| serological data not available | |  |  |
| 8 cases |  |  |  |

*Cutoff titer: 1:20 for all antigen preparations
